# Chronic disease prevalence from Italian administrative databases in the VALORE project: a validation through comparison of population estimates with general practice databases and national survey

**DOI:** 10.1186/1471-2458-13-15

**Published:** 2013-01-09

**Authors:** Rosa Gini, Paolo Francesconi, Giampiero Mazzaglia, Iacopo Cricelli, Alessandro Pasqua, Pietro Gallina, Salvatore Brugaletta, Daniele Donato, Andrea Donatini, Alessandro Marini, Carlo Zocchetti, Claudio Cricelli, Gianfranco Damiani, Mariadonata Bellentani, Miriam CJM Sturkenboom, Martijn J Schuemie

**Affiliations:** 1, Agenzia regionale di sanità della Toscana, Via Pietro Dazzi 1, 50141 Florence, Italy; 2, Department of Medical Informatics, Erasmus Medical Center, Dr. Molewaterplein 50, 3015 GE Rotterdam, The Netherlands; 3, Società italiana di medicina generale, Via del Pignoncino, 9-11, 50142 Florence, Italy; 4, Genomedics, Via Sestese 61, 50141 Florence, Italy; 5, ULSS 16 Padova, Via Enrico Degli Scrovegni 14, 35131 Padua, Italy; 6, ASP 7 Ragusa, Piazza Igea 1, 97100 Ragusa, Italy; 7, Assessorato Politiche per la Salute, Viale Aldo Moro 21, 40127 Bologna, Italy; 8, Zona Territoriale Senigallia, Via Piero della Francesca 14; 9, Regione Lombardia, Piazza Città di Lombardia 1, 20124 Milan, Italy; 10, Università Cattolica del Sacro Cuore, Largo Francesco Vito 1, 00198 Rome, Italy; 11, Agenzia Nazionale per il Servizi Sanitari Regionali, Via Puglie 23, 00187 Rome, Italy

**Keywords:** Prevalence, Chronic disease, Validation studies, Data reuse

## Abstract

**Background:**

Administrative databases are widely available and have been extensively used to provide estimates of chronic disease prevalence for the purpose of surveillance of both geographical and temporal trends. There are, however, other sources of data available, such as medical records from primary care and national surveys. In this paper we compare disease prevalence estimates obtained from these three different data sources.

**Methods:**

Data from general practitioners (GP) and administrative transactions for health services were collected from five Italian regions (Veneto, Emilia Romagna, Tuscany, Marche and Sicily) belonging to all the three macroareas of the country (North, Center, South). Crude prevalence estimates were calculated by data source and region for diabetes, ischaemic heart disease, heart failure and chronic obstructive pulmonary disease (COPD). For diabetes and COPD, prevalence estimates were also obtained from a national health survey. When necessary, estimates were adjusted for completeness of data ascertainment.

**Results:**

Crude prevalence estimates of diabetes in administrative databases (range: from 4.8% to 7.1%) were lower than corresponding GP (6.2%-8.5%) and survey-based estimates (5.1%-7.5%). Geographical trends were similar in the three sources and estimates based on treatment were the same, while estimates adjusted for completeness of ascertainment (6.1%-8.8%) were slightly higher. For ischaemic heart disease administrative and GP data sources were fairly consistent, with prevalence ranging from 3.7% to 4.7% and from 3.3% to 4.9%, respectively. In the case of heart failure administrative estimates were consistently higher than GPs’ estimates in all five regions, the highest difference being 1.4% vs 1.1%. For COPD the estimates from administrative data, ranging from 3.1% to 5.2%, fell into the confidence interval of the Survey estimates in four regions, but failed to detect the higher prevalence in the most Southern region (4.0% in administrative data vs 6.8% in survey data). The prevalence estimates for COPD from GP data were consistently higher than the corresponding estimates from the other two sources.

**Conclusion:**

This study supports the use of data from Italian administrative databases to estimate geographic differences in population prevalence of ischaemic heart disease, treated diabetes, diabetes mellitus and heart failure. The algorithm for COPD used in this study requires further refinement.

## Background

Administrative healthcare data are collected by privately owned health maintenance organisations or government-run institutions for managerial reasons. Due to differences in healthcare systems, the content of the administrative data may vary from country to country. They may contain records collected at hospital discharge, during encounters with the general practitioner (GP) or specialist, at drug prescription or dispensation, or upon request for, or conduct of, a diagnostic analysis or procedure. The content also depends on the choices of the organisation: data may or may not contain diagnosis codes; drug prescriptions may or may not contain indication of use, data from laboratories may or may not contain the actual result.

Secondary use of administrative healthcare data has been increasing over the years, including the provision of prevalence estimates for chronic diseases [1], such as diabetes mellitus, chronic obstructive pulmonary disease (COPD), hypertension, ischaemic heart disease, cerebrovascular disease, and depression. Case finding and ascertainment algorithms are tailored to the structure and type of information that is captured in the specific administrative database. Sensitivity and specificity of such algorithms are conditioned on distinguishing features such as presence of drug dispensing as well as sources for diagnostic codes. The Canadian [2], Swedish [3] and Medicare [4] administrative databases, for example, contain diagnosis codes from hospitalization episodes as well as from outpatient care, hence enriching the data for estimation of chronic disease prevalence. In settings where outpatient care diagnoses are not available, other solutions have been explored. For instance, in Luxembourg and France, where only drug prescriptions are available, diabetes could be identified in treated patients by analysing the volume of prescriptions for anti-diabetic drugs [5].

Observational studies based on administrative databases need careful validation of the algorithms they rely on in order to provide sound epidemiologic research [6]. Validation of chronic disease case ascertainment algorithms has been performed either through direct [7] or indirect [8] clinical assessment or through individual record linkage with other electronic data sources, such as disease registries [9] or health surveys [4,10]. When individual record linkage with non-administrative data sources was not feasible, the performance of the algorithms has been inferred though external comparison with prevalence estimates obtained from health surveys [5,11].

Italy has a tax-based, universal coverage national health system organised in three levels: national; regional (21 regions); and local (on average 10 local health units per region) [12]. Administrative data on healthcare reimbursed by the system, such as inpatient care and drug dispensations, are routinely collected by local health units and, in some regions, sent to the regional level. Transmission to the national level is obligatory, and a common data model for data transmission is mandated by law on a national level. Before data are sent to the national level, however, unique personal identifiers are removed, hence record-linkage cannot be performed outside a single region. The Italian administrative databases therefore form a virtual national information system, with homogeneous data collected at the local level. Actual databases allowing record-linkage only exist up to the regional level.

Data on diagnosis collected in outpatient settings are not part of the Italian administrative databases, therefore algorithms for case ascertainment developed in other countries that make use of this information cannot be applied to the Italian situation. Several studies have investigated the comparison between chronic disease prevalence estimates from Italian administrative data and other Italian data sources [11,13]. However studies to date were only performed within local or regional databases. Capture–recapture technique, a more sophisticated analysis aimed at estimating a suspected underascertainment when more than two lists of cases are available [14,15], was applied as well in estimating diabetes prevalence from administrative databases, in specific geographic areas of the country [16,17]. In light of the strong difference in health and healthcare quality across Italy [18] it is relevant to understand whether administrative databases can support chronic disease prevalence surveillance in different areas of the country.

In 2010 the Italian national project VALORE was launched aimed at assessing quality of care for chronic diseases in five different Italian regions, based on secondary use of data from administrative databases. In this study we describe prevalence estimates for diabetes mellitus, heart failure, ischaemic heart disease and COPD from these data and compare the estimates with prevalence estimates obtained from a national GP electronic medical record database and, where possible, from a national health survey.

## Methods

### Setting

The five regions which contributed data to the VALORE study were: Veneto (A, Northern Italy), Emilia Romagna (B, Northern Italy), Tuscany (C, Central Italy), Marche (D, Central Italy) and Sicily (E, Southern Italy). The following data files were used in the VALORE project: 

**Hospital discharge records** with one main and five secondary diagnoses coded using the International Classification of Diseases, Ninth Revision, Clinical Modification (ICD9CM);

**Drug dispensing records** coded using Anatomic Therapeutic Chemical (ATC) codes for drug classification; the ATC system is the drug classification system adopted by the World Health Organization [19];

**Disease-specific exemptions from copayment to health care** coded using ICD9CM;

**Inhabitant Registry (IR)** with demographic information (birthyear, gender) and identifier of the GP in charge.

In each region, record-linkage within and between data files was done deterministically with a unique coded personal identifier. Region B could not provide the file of exemptions from copayment. For organizational reasons, the regions participating in the VALORE project did not provide administrative data of the whole regional population, but only of specific geographical subareas. In each region raw data were extracted from the local data files and sent via File Transfer Protocol (FTP) to a single data management center, after anonymization of the coded personal identifier. A standardized automated routine was developed in Stata 9.2 to apply the case ascertainment algorithm and to calculate the prevalence estimates. Each regional sample consisted of all inhabitants registered in the selected geographical subareas and alive at the index date (January 1st 2009).

### Case ascertainment

The case finding and ascertainment algorithms that were used to detect the specific diseases are shown in Table 1. Regional administrative databases link Hospital discharge records (HOSP), Drug dispensation records (DRUG), and Disease-specific exemptions (EXE) from 2003 to 2008, and a patient was classified as having the selected disease if at least one of the corresponding conditions listed in Table 1 were met, i.e. condition 1 OR condition 2 OR condition 3. For 93% of the population, the full six years of follow-up data were available and were included in the analysis.

**Table 1:** Case ascertainment algorithms for diabetes, ischaemic heart disease, heart failure and COPD

**Disease**	**Administrative data**			**GP data**
	HOSP	DRUG	EXE	PROBLEM
	(ICD9CM)^+^	(ATC)^++^	(ICD9CM)	(ICD9CM)
Diabetes mellitus	250*	A10	250	250*
Treated diabetes		A10		250* AND
				A10^++++^
Ischaemic heart disease	410-*414*	C01DA	414	410-*414*
Heart failure	428*, 40201,	-	428	428*, 40201,
	40211, 40291,			40211, 40291,
	40401, 40403,			40401, 40403,
	40411, 40413,			40411, 40413,
	40491, 40493			40491, 40493
COPD	490*-492*,	R0^+++^	-	490*-492*,
	494*, 496*			494*, 496*

Algorithms for case ascertainment of diabetes, ischaemic heart disease, heart failure, and chronic obstructive pulmonary disease (COPD), respectively from regional administrative databases and from GP databases. Regional administrative databases link Hospital discharge records (HOSP), Drug dispensation records (DRUG), and Disease-specific exemptions (EXE) from 2003 to 2008, and a patient was classified as having the selected disease if at least one of the listed conditions were met, ie condition 1 OR condition 2 OR condition 3. GP databases were queried in the PROBLEM field of the clinical database, where diagnosis are coded.

^+^Either in main or in one of the secondary diagnoses.

^++^At least two dispensations in different dates in a single year.

^+++^A specific algorithm involving number, heterogeneity of ATC codes and time span of dispensations is used, see [21].

^++++^Patients having at least 2 prescriptions in one of the previous 2 years.

Diverse algorithms for diabetes, COPD and ischaemic heart disease case ascertainment from Italian administrative databases have been previously described in the literature. Those published in Simonato et al. [20] were the result of a workgoup involving two Italian scientific associations of epidemiologists and biostatisticians, and were therefore adopted. However, to deal with a previously reported issue of lack of sensitivity, the algorithm for COPD was enriched with drug dispensing data [21]. In addition we also calculated a prevalence estimate of diabetes based on anti-diabetic treatment alone. The heart failure algorithm was defined in the VALORE project.

### Comparison data

The Health Search Database (HSD) collects electronic medical record data from a network of Italian GPs who are members of the Italian College of General Practitioners [22]. The GPs participating in HSD all use the same information software, in which they record demographic information, visits and referrals, diagnoses (both in free text and ICD9CM codes), drug prescriptions and clinical information. For this study, data from 199 GPs practicing in one of the five regions of the VALORE project were used. The study population comprised patients aged 16-95 who had been enrolled for at least two years and were alive on 1st January 2009. Prevalence estimates were calculated based on the number of patients enrolled with the GPs at the index date (January 1st 2009) as denominator. The numerator represented all cases with specific diseases as ascertained through a query in the PROBLEM field of the clinical database, where diagnoses are coded. The diagnosis codes are shown in Table 1.

The Italian National Health Survey is conducted every five years by the National Institute of Statistics (ISTAT). In addition, there is a yearly survey that captures relevant health-related issues, and in particular diabetes and COPD. The 2008 survey in the five regions of the VALORE study comprised 11,656 people aged 16 years and above. The survey sample was extracted according to a two-stage weighted cluster sampling design (first level: municipalities; second level: families). Answers to two questions were used for this study: *Are you affected by one or more of the following chronic diseases? Diabetes (Yes/No) Chronic bronchitis, emphisema, repiratory failure (Yes/No)*. Questions about heart failure and ischaemic heart disease were not asked.

### Data analysis

Administrative data provided estimates of prevalence in three different ways: (1) analysis of distribution of prevalence per GP practices; (2) pooled analysis; (3) capture-recapture analysis. While (2) and (3) provided estimates that could be compared with the regional estimates from the other two data sources, it must be noted that as the population sample from each regional population is not random, but rather geographically restricted, it was expected that the comparison was biased. The rationale for analysis (1) was therefore the following: as both administrative and GP data could be aggregated per practice and as practices could be considered to be (non randomly) sampled from the same population of regional practices, if the two measurement techniques (administrative versus GP data) did measure in fact the same population parameter it was expected that the pairs of regional distributions overlapped and distributions within the same region were more similar to each other than distributions within the same data source.

All the analyses refer to the population aged ≥ 16 years, both male and females, although in the GP database ages above 95 were truncated. Sex and age distribution of each regional sample were computed. The percentage of the regional population covered by the sample was estimated by dividing its number by the estimates of the regional population according to the National Institute of Statistics [23]. Prevalence was estimated as the total number of existing cases divided by the number of subjects in the sample. Every adult Italian inhabitant is entitled to choose a GP, and GPs may accept a maximum of 1,500 patients [12]. For each GP the population registered with that GP at the index date was calculated and used as denominator for the prevalence estimates. Median and interquartile (IQ) range of this distribution was computed in each regional sample. To avoid spurious results the disease prevalence per GP practice was computed only for those practices who had at least 300 people enrolled and at least 4 patients with the disease.

In the Health Search Database the same prevalence measures were estimated, the sample being the number of inhabitants in charge of the GPs of HSD at the index date.

From the National Health Survey the variable of the first-level sampling design (municipalities) was not available, hence simple weighted analysis was performed, with probability weight attributed to each individual.

Finally, to ascertain the degree of completeness in capturing diabetes cases from administrative data, a capture-recapture analysis was performed. Log-linear models were estimated by sequentially incorporating pair-wise dependency between sources, and model selection was based on the Akaike information criterion (AIC) criterion. This was not done for region B, where only two sources of data were available, since independence between two data sources could not be assumed [15].

All analyses were performed with Stata 9.2.

### Ethical approval

No identifiable human data were used for this study. The dataset used in the study is not openly available. Permission to use non-identifiable, individual data extracted from administrative databases for the VALORE project was granted by ULSS 16 Padova, ASP 7 Ragusa, Assessorato Politiche per la Salute Emilia Romagna, Zona Territoriale Senigallia, which are responsible for the use of the data of the corresponding populations. Agenzia regionale di sanità della Toscana is enabled by a regional law to use Tuscan data for research purposes. Approval for use of encrypted and aggregated data from the HSD was also obtained from the Italian College of General Practitioners. Data from the National Health Survey are openly available from ISTAT.

## Results

The subpopulations whose data were collected covered a percentage of the total population of the regions, as shown in Table 2.

**Table 2:** Subpopulations covered by administrative, GP and survey data

**Region**	**Population aged 16+ (millions)**	**Administrative data coverage**			**GP data coverage**			**Survey data coverage**	
		N GPs	N sample	% pop	N GPs	N sample	% pop	N sample	% pop
A	4.2	140	167,805	4.0	51	70,301	1.7	2,551	0.06
B	3.7	625	840,546	22.5	41	60,59	1.6	2,317	0.06
C	3.2	511	498,084	15.5	29	36,908	1.1	2,410	0.07
D	1.3	57	63,125	4.7	18	24,912	1.8	1,728	0.13
E	4.2	231	264,902	6.3	60	84,483	2.0	2,650	0.06

Characteristics of the subpopulations of each region covered, respectively, by administrative, GP and National Health Survey data. Data on general population from the Italian National Institute of Statistics. Analysis is restricted to inhabitants aged 16+ and, for GP data, 16-95.

**Figure 1:**
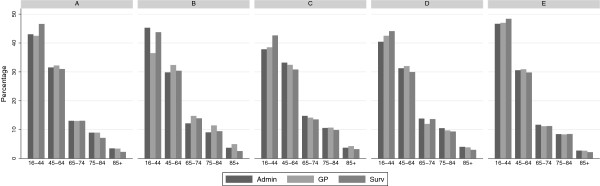
**Age distribution in each region from each data source.** Age distribution in each region of the sample extracted from administrative databases (Admin), of the sample extracted from clinical data collected by GPs participating to the Health Search Database (GP) and of the sample participating to the National Health Survey (Surv).

The age distribution of the three population samples in the five regions is shown in Figure 1. There were some differences in age distribution between the populations from the different sources (see Figure 1).

**Figure 2:**
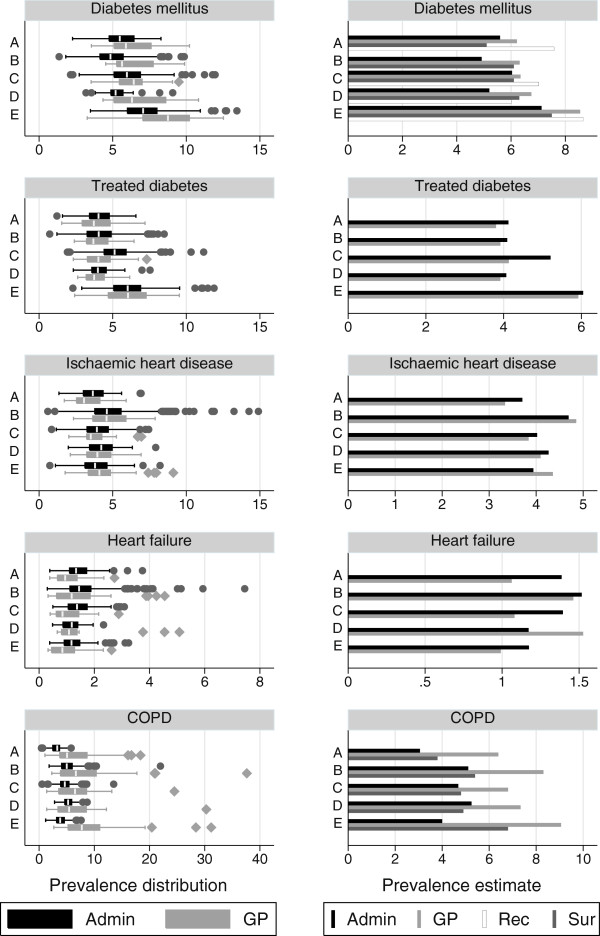
**Prevalence estimates for diabetes mellitus, treated diabetes, ischaemic heart disease, heart failure and COPD from each data source.** Crude prevalence estimates for diabetes mellitus, treated diabetes, ischaemic heart disease, heart failure and COPD in 5 Italian regions, according to administrative data (Admin) and clinical GP data (GP) and, for diabetes and COPD only, the National Health Survey (Sur). For diabetes mellitus estimates from administrative data adjusted for ascertainment are also presented (Rec). On the left column prevalence is represented by box plots of the distribution of the disease prevalence in GP practices: the central line is the median value, the box covers the interquartile range, while wiskers range from a minimum to a maximum value except for some observations which are detected as outliers and are representes as single dots or diamonds; comparison is only betweem GP and Admin data sources. On the right column prevalence is represented as global estimate. Date: 1 January 2009 Population: male and females, aged 16+.

The prevalence estimates in the five regions from the three sources are shown in Figure 2 and Table 3. Administrative data underestimated the prevalence of diabetes as compared to both GP and survey estimates across most regions although differences were often barely or non significant and the increasing North-South trend could be consistently observed in the three sources. Adjustement for underascertainment led to higher estimates with respect to both GP and Survey figures, except in one region. The width of the interquartile range (IQ) of the practice-level estimates was higher in GP data than in administrative databases. When prevalence of diabetes was estimated based only on diabetes treatment, the prevalence estimates obtained from GP data were fairly consistent with those obtained from administrative data in all regions; the width of the IQ range of the practice estimates was similar between GP data and administrative data in all regions.

**Table 3:** Table of prevalence estimates for diabetes mellitus, treated diabetes, ischaemic heart disease, heart failure and COPD from each data source

		**A**		**B**		**C**		**D**		**E**	
**Disease**	**Data source**	**Mean**	**Median**	**Mean**	**Median**	**Mean**	**Median**	**Mean**	**Median**	**Mean**	**Median**
	**source**	**(95% CI)**	**(IQ Range)**	**(95% CI)**	**(IQ Range)**	**(95% CI)**	**(IQ Range)**	**(95% CI)**	**(IQ Range)**	**(95% CI)**	**(IQ Range)**
Diabetes mellitus	Admin	5.6 (5.5-5.7)	5.5 (4.8-6.4)	4.9 (4.9-5.0)	4.8 (4.1-5.7)	6.0 (6.0-6.1)	6.0 (5.1-6.9)	5.2 (5.0-5.4)	5.2 (4.9-5.7)	7.1 (7.0-7.2)	7.1 (6.0-8.0)
	GP	6.2 (6.0-6.4)	5.9 (5.1-7.6)	6.3 (6.1-6.5)	5.6 (5.3-7.7)	6.3 (6.1-6.6)	6.4 (5.5-7.0)	6.7 (6.4-7.1)	6.3 (5.1-8.6)	8.5 (8.4-8.7)	8.8 (7.0-10.2)
	Survey	5.1 (4.2-6.0)		6.1 (5.1-7.1)		6.1 (5.1-7.1)		6.3 (5.2-7.4)		7.5 (6.4-8.5)	
	Admin-Recap	7.6 (7.1-8.2)				7.0 (6.8-7.3)		6.0 (5.8-6.4)		8.7 (8.4-9.0)	
Treated diabetes	Admin	4.1 (4.0-4.2)	4.0 (3.4-4.8)	4.1 (4.0-4.1)	4.0 (3.3-4.9)	5.2 (5.1-5.3)	5.1 (4.4-5.9)	4.1 (3.9-4.2)	4.0 (3.6-4.6)	6.0 (6.0-6.1)	6.0 (5.1-6.9)
	GP	3.8 (3.7-3.9)	3.7 (2.9-4.8)	3.9 (3.8-4.1)	3.7 (3.2-4.7)	4.1 (3.9-4.3)	4.0 (3.3-4.8)	3.9 (3.7-4.2)	3.7 (3.2-4.4)	5.9 (5.8-6.1)	6.0 (4.7-7.3)
Ischaemic heart disease	Admin	3.7 (3.6-3.8)	3.6 (3.0-4.3)	4.7 (4.6-4.7)	4.6 (3.8-5.6)	4.0 (4.0-4.1)	3.9 (3.3-4.7)	4.3 (4.1-4.4)	4.2 (3.4-5.0)	3.9 (3.9-4.0)	3.8 (3.1-4.6)
	GP	3.3 (3.2-3.5)	3.1 (2.5-4.1)	4.9 (4.7-5.0)	4.6 (3.6-5.9)	3.8 (3.6-4.0)	3.5 (3.2-4.3)	4.1 (3.8-4.3)	4.0 (3.2-4.9)	4.4 (4.2-4.5)	4.1 (3.3-4.8)
Heart failure	Admin	1.4 (1.3-1.4)	1.3 (1.1-1.7)	1.5 (1.5-1.5)	1.4 (1.1-1.9)	1.4 (1.4-1.4)	1.4 (1.1-1.7)	1.2 (1.1-1.3)	1.2 (0.9-1.4)	1.2 (1.1-1.2)	1.2 (0.9-1.5)
	GP	1.1 (1.0-1.1)	0.9 (0.7-1.4)	1.5 (1.4-1.6)	1.2 (0.6-1.8)	1.1 (1.0-1.2)	0.8 (0.6-1.4)	1.5 (1.4-1.7)	1.1 (0.8-1.4)	1.0 (0.9-1.1)	0.8 (0.5-1.3)
COPD	Admin	3.1 (3.0-3.1)	3.3 (2.5-3.7)	5.1 (5.1-5.2)	5.0 (4.1-6.0)	4.7 (4.6-4.7)	4.7 (3.9-5.4)	5.2 (5.1-5.4)	5.2 (4.7-5.9)	4.0 (3.9-4.1)	3.8 (3.2-4.6)
	GP	6.4 (6.2-6.6)	5.1 (3.8-8.7)	8.3 (8.1-8.5)	6.7 (3.9-10.3)	6.8 (6.5-7.1)	6.5 (3.6-8.6)	7.3 (7.0-7.7)	5.4 (3.4-8.6)	9.1 (8.9-9.2)	7.7 (5.3-11.0)
	Survey	3.8 (3.0-4.6)		5.4 (4.4-6.4)		4.8 (3.9-5.7)		4.9 (3.8-5.9)		6.8 (5.8-7.8)	

Crude prevalence estimates for diabetes mellitus, treated diabetes, ischaemic heart disease, heart failure and COPD in 5 Italian regions, according to administrative data (Admin) and clinical GP data (GP). For diabetes and COPD only: crude prevalence estimated from the National Health Survey (Surv). For diabetes only: prevalence estimates from administrative data adjusted for estimated completeness of ascertainment (Admin-Recap). Prevalence estimated both as global percentage with 95% confidence interval and, for Admin and GP only, as median with interquantile range of the distribution of prevalence in GP practices. Date: 1 January 2009. Population: male and females, aged 16-95 in GP and 16+ in the other sources.

The prevalence of ischaemic heart disease, as estimated using administrative data, was similar to that estimated using GP data, prevalence ranging from 3.3% (region A, source GP) to 4.9% (region B, source GP). The width of the IQ range was similar between GP data and administrative data in all regions.

**Figure 3:**
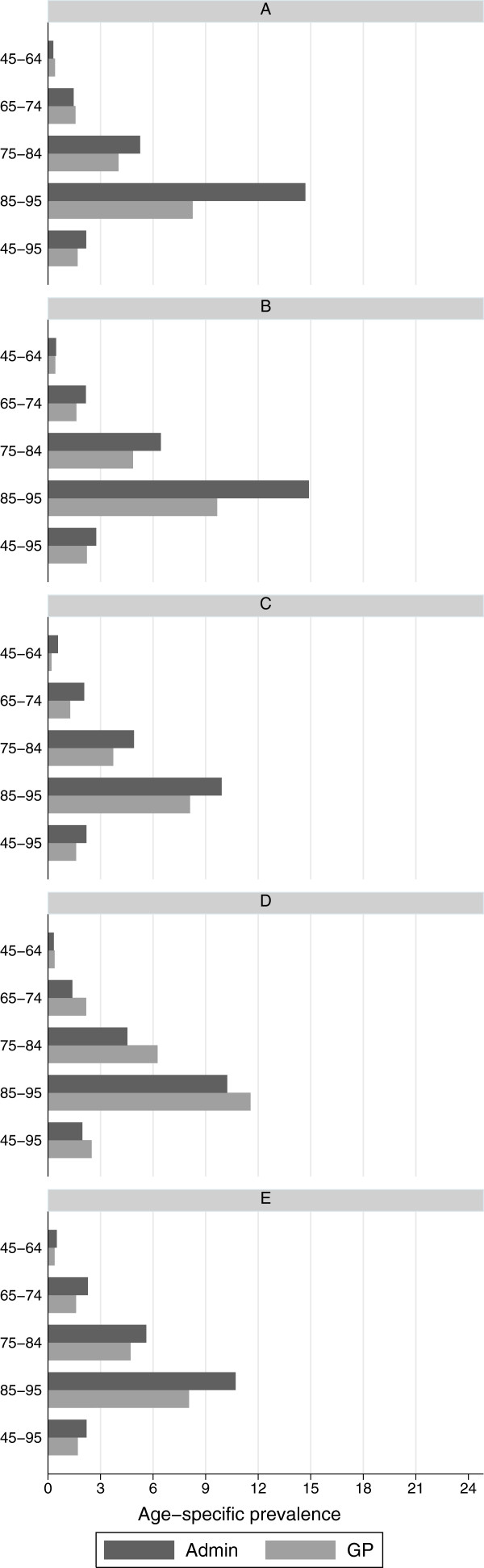
**Age-specific prevalence of heart failure.** Age-specific prevalence of heart failure in 5 Italian regions, according to administrative data (Admin) and clinical GP data (GP). Date: 1 January 2009 Population: male and females, aged 16+.

The prevalence estimates for heart failure were lower in GP data than in administrative data in three regions, with the highest difference (1.1% vs 1.4%) observed in both regions A and C, where significativeness was observed as well. According to age-specific prevalence estimates shown in Figure 3, the difference in estimates were increasing with age. The width of IQ range of the practice estimates was higher for GP data in three regions.

The prevalence estimates from administrative data for COPD in regions A to D fell within the confidence interval of the survey estimates and ranged from from 3.8% (region A) to 4.9% (region D), but failed to detect the higher prevalence in Region E (4.0% in administrative data vs 6.8% in survey data). The estimates from GP data were consistently higher, ranging from 6.4% (region A) to 9.1% (region E), and detected the same increased prevalence in region E. The width of IQ range of the practice estimates was much higher for GP data.

In all diseases the width of the IQ range of the practice estimates were fairly consistent across regions in administrative data. Mean and medians were pretty close in both sources and all regions, and, except for outliers, distributions were rather symmetric, although in GP data data the distribution was slightly skewed to the right for diabetes and COPD.

## Discussion

Overall, differences in prevalence estimates among the different sources in a region were lower than the differences between regions, and differences observed among regions were similar across data sources. The fact that independent sources of data showed consistent values across different regions supports the claim that they correspond to actual population measures. In case systematic differences were observed, they could be interpreted as being due to differences in data collection and associated to demographic and disease characteristics. This provides evidence that administrative data actually measure a population phenomenon that can be interpreted and supports the use of administrative data for surveillance of geographical trends of the diseases in study, with the possible exception of COPD.

In the case of diabetes mellitus, the observed concordance between estimates from GP data and from survey data confirms previous reports [24,25]. Estimates from GP data were systematically higher than estimates from administrative data. According to reports from other countries [26], the difference is likely to be due to the proportion of patients who, although being diagnosed with diabetes to the knowledge of their GPs, have mild or well-controlled disease and thus have never had either a hospital admission or a prescription for antidiabetic drugs, and have not received an exemption from copayment of diabetes-related healthcare, therefore escaping the algorithm for administrative databases. Indeed, when the subset of patients undergoing therapy with antidiabetics in the previous two years were extracted from both administrative and GP data sources, the pairs of prevalence estimates almost coincided in all of the regions, with one exception. Estimates adjusted for completeness of ascertainment, on the other hand, provided slightly higher estimates, a finding consistent with a previous study with similar data in another Italian area [16].

Ischaemic heart disease being congruently estimated by administrative and GP data in all of the regions is an unexpected finding. Angina, a less severe form of the disease, does not lead *per se* to a hospital admission, and few cases (less than 5%) are detected by the registry of exemptions from copayment. As around 30% of cases are detected only by dispensings of nitrates (data not shown), we observe that nitrates therapy is probably specific in detecting cohorts bearing this condition, as otherwise data would have been less consistent across regions in matching the diagnosis-based figures from GP clinical databases.

Heart failure was underestimated by GP data, although non significantly in the majority of the regions. Underestimation was highest in the oldest age band available in both data sources (85-95), where the prevalence is highest. This is consistent with the hypothesis that GPs belonging to HSD might occasionally perform less accurate data collection when visiting patients at home [27] or in residential care [22], or consider heart failure as a complication of other underlying conditions, such as ischaemic heart disease, rather than as a disease of its own. This would imply that the population detected by administrative data had a more severe form of the disease and was more often affected by disability. Another possibility is that administrative database overestimate prevalence because of lack of specificity of the case ascertainment algorithm. Indeed, according to a recent review of validated algorithms for case ascertainment of heart failure [28], algorithms using secondary discharge diagnosis showed lower positive predictive value (PPV) in several countries.

For COPD administrative data failed to detect the differences between regions that the other two sources consistently measured. Ascertainment of COPD from administrative sources has been shown to be challenging in other studies [29,30]. In this case, the algorithm detected a particular pattern of drug prescriptions, combining duration, intensity and ATC class, that had been identified through a consensus process in a group of experts that was reported in Anecchino et al. [21]. Although the pattern was specifically meant to avoid misclassification (e.g., with respect to asthma), it is possible that the conclusions of the study were in fact specific for the geographic area where the experts worked.

In light of the limitations of the sampling design of our study, the overall good agreement with other data sources supports *a fortiori* validity of chronic disease surveillance using administrative data in the regions that were involved in the study. However, support for external validity of our results needs to be discussed. Althought we only collected data from few geographical areas, the same administrative data are available for the whole national population. We are in fact not claiming that administrative data from few geographically sparse areas can be used to estimate *national* prevalence of chronic diseases, but rather that administrative data seem to be consistently able to detect prevalence of some chronic diseases *around the area they were extracted from*. Our positive findings (treated diabetes, ischaemic heart disease, heart failure) are indeed probably due to the fact that typical health consumption patterns of such chronic patients are similar across regions. On the assumption that regions of the same macroarea of the country (North, Center, South) are similar the one to the other to this respect, our data support the claim that estimates relying on the same algorithms should prove to be similarly effective. However, in some specific critical areas of the country where incomplete administrative data collection is suspected, a local evaluation is recommended.

Cohorts can be selected from administrative databases to perform population-based studies on patients with chronic diseases through further record-linkage with the same databases. This study cannot provide analytical tools to assess the limitations of the findings of such studies. However, no evidence emerges for major bias, except in the case of COPD, where regional differences with the other data sources are likely to be due to differences in the characteristics of the corresponding local cohorts.

### Limitations

The first limitation of studies that make secondary use of existing healthcare data sources is that only prevalence of diagnosed cases is taken into account, and underestimation of actual population prevalence cannot be estimated [31].

An implicit assumption of both crude and adjusted rate estimation from administrative databases performed in this study was that PPV of the case detection was 100%, an assumption that we could not verify and that is not to be taken for granted, when, for instance, secondary discharge diagnosis or drug utilisation with no indication is used as a source of case ascertainment. Ecological validation studies cannot directly resolve this issue, as consistent ecological estimates between a data source and a reference gold standard might as well be due to coincidental inclusion of false positive and exclusion of false negative cases. Only validation studies perfomed using individual-level comparison with a gold standard could assess PPV and sensitivity.

## Conclusion

This study supports the use of data from Italian administrative databases to estimate geographic differences in population prevalence of ischaemic heart disease, treated diabetes, diabetes mellitus and heart failure. The algorithm for COPD used in this study requires further refinement.

## Abbreviations

AIC: Akaike information criterion; ATC: Anatomic, Therapeutic, Chemical Classification System; COPD: Chronic Obstructive Pulmonary Disease; FTP: File Transfer Protocol; GP: General Practitioner; HSD: Health Search Database; ICD9CM: International Classification of Diseases Ninth Revision Clinical Modification; IQ: Interquartile range; IR: Inhabitant Registry; ISTAT: National Institute of Statistics.

## Competing interests

The authors declare that they have no competing interests.

## Author’s contributions

RG conceived of the study, participated in its design and coordination, performed data management and data analysis and drafted the manuscript. PF conceived of the study, participated in its design and coordination with responsability of the Tuscany data and helped to draft the manuscript. GM conceived of the study, participated in its design and coordination and helped to draft the manuscript. IC participated in the study design and coordination. AP provided data management and data analysis of the Health Search data. PG participated in the study design and coordination and helped to draft the manuscript. SB participated in the study coordination with responsability of the Sicily data and helped to draft the manuscript. DD participated in the study coordination with responsability of the Veneto data. AD. participated in the study coordination with responsability of the Emilia Romagna data and helped to draft the manuscript. AM participated in the study coordination with responsability of the Marche data. CZ participated in the study coordination. CC participated in the study coordination with responsability of the Health Search data. GD participated in the study design and helped to draft the manuscript. MB participated in the study design and coordination. MCJMS participated in the study design and reviewed the drafts of the manuscript. MJS participated in the study design and reviewed the drafts of the manuscript. All authors read and approved the final manuscript.

## Pre-publication history

The pre-publication history for this paper can be accessed here:

http://www.biomedcentral.com/1471-2458/13/15/prepub
